# Application of isolated *Lactobacillus sakei* and *Staphylococcus xylosus* strains as a probiotic starter culture during the industrial manufacture of Tunisian dry‐fermented sausages

**DOI:** 10.1002/fsn3.1711

**Published:** 2020-07-03

**Authors:** Afef Najjari, Mohamed Boumaiza, Sana Jaballah, Abdelatif Boudabous, Hadda‐Imene Ouzari

**Affiliations:** ^1^ Faculté des Sciences de Tunis LR03ES03 Microorganismes et Biomolécules Actives Université de Tunis El Manar Tunis Tunisia

**Keywords:** dry‐fermented sausages, *Lactobacillus sakei*, nutritional quality, physicochemical quality, sensory quality, *Staphylococcus xylosus*

## Abstract

For decades, lactic acid bacteria has been isolated and selected to be used as starter cultures in meat fermentation for standardization and management of quality of dry‐fermented sausage which constitute a considerable challenge. The aim of this study was to evaluate the effect of *Lactobacillus sakei* strains, isolated from different origins, on qualities of dry‐fermented sausages. These last, manufactured with different combinations of starter cultures (*L. sakei* + *Staphylococcus xylosus*), were ripened, using the same raw materials and conditions, for 45 days. Samples were collected during this period, and microbiological, physicochemical, fatty acid profile, and sensorial analyses determined. Lactic acid bacteria were the dominant flora during ripening. A desirable PUFA/SFA ratio, corresponding to 1:1.7 (0.6), was detected after 24 days of maturation in sausages inoculated by *L. sakei* BMG 95 and *S. xylosus*. Sensory analysis showed that fermented sausages manufactured with *L. sakei* and *S. xylosus* had a more desirable odor, flavor, and texture and consequently were preferred overall. In particular, sensory panellists preferred sausages produced with either *L. sakei* 23K or *L. sakei* BMG 95 when compared to fermented sausage produced with a commercial starter or no starter at all.

## Graphical abstract

Dry‐fermented sausage samples inoculated with *Lactobacillus sakei* BMG 95 showed best physicochemical, nutritional, and sensory satisfactory results in comparison with the commercial starter. This strain, isolated from anchovies, was able to produce a fermented sausage with similar/or better quality as sausages fermented with the commercial starter.

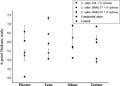

## 1 INTRODUCTION

Fermentation is one of the oldest preservation methods known to human society and in particular to people living in Mediterranean countries (Benito et al., 2007; Lucke, 1998; Oliveira, Ferreira, Magalhães, & Teixeira, 2018; Zagorec & Champomier‐Vergès, 2017; Zeuthen, 1995). Fermentative processes have primarily been used for the conservation of food, using bacteria that convert sugars present in the raw product into products such as lactic acid. The resulting reduction in pH leads to impaired development of a range of microorganisms and thus contributes to the assurance of a microbiologically healthy product. Fermentation of sausage utilizing indigenous microflora still exists (Klingberg, Axelsson, Naterstad, Elsser, & Budde, 2005; Rantsiou et al., 2005). For example, in Greece, Italy, and Spain, efforts are still dedicated to the study of traditional production methods (Comi et al., 2004). In such products, lactic acid bacteria (LAB) and coagulase‐negative cocci (CNC) are the most active of the indigenous microorganisms involved (Hammes & Hertel, 1998; Lucke, 2000). However, with the increasing interest in biological preservation techniques, the application of LAB as starter and/or protective cultures is attracting growing interest (Devleighere, Vermeiren, & Debevere, 2004; Holzapfel, Geisen, & Schillinger, 1995; Lucke, 2000; Vermeiren, Delieghere, & Dedevere, 2004). In particular, the use of starter cultures for sausage production is becoming increasingly necessary to improve safety, to standardize product properties including consistent flavor and color, and to shorten ripening times (Laranjo, Potes, & Elias, 2019; Leistner, 1995, 2000). The commonly favored microorganisms used as starters tend to be species of *Lactobacillus, Pediococcus, Micrococcus,* and *Staphylococcus* (Hammes & Knauf, 1994). However, fungal starters have also been used to ferment such meat products (Toledo, Selgas, Casas, Ordonez, & Gracia, 1997; Trigueros, Gracia, Casas, Ordonez, & Selgas, 1995). In contrast, some processors have endeavored to accelerate the ripening process by using proteases and lipases from a range of sources (Andersen & Østdal, 1995; Blom et al., 1996; Bruna, Fernandez, Hierro, Ordonez, & Hoz, 2000a, 2000b; Zapelena, Astiasaran, & Bello, 1999). Several studies have examined the effect of using combinations of starter cultures for the ripening of fermented sausage (Candogan, Kartika, Wardlaw, & Acton, 2008; Kenneally, Schwarz, Fransen, & Arendt, 1998; Marco, Navarro, & Flores, 2008; Marcos, Teresa, Guardia, & Garriga, 2007). In particular, staphylococci and micrococci have been used in combination with LAB due to their ability to contribute to flavor, mainly through lipolytic activities, and also due to their abilities to reduce nitrate and produce catalase which could contribute also to fix and obtain the typical color of the final product (Lucke, 1986; Nychas & Arkoudelos, 1990).

The manufacture of dry‐fermented sausage is considered to occur in three phases: formulation (mixing of ingredients), fermentation, and ripening/drying (Fernandez, Ordonez, Bruna, Herranz, & Hoz, 2000). During the ripening of dry‐fermented sausage, microbiological, biochemical, and sensorial changes take place that are closely related to the activity of the dominant microorganisms present in the sausage matrix. The LAB most frequently isolated from dry sausage are *Lactobacillus sakei*, *Lactobacillus curvatus,* and *Lactobacillus plantarum* (Hammes, 1990; Schillinger & Lucke, 1987). *Lactobacillus sakei* is the bacterial species most frequently preferred in Western Europe as a meat starter culture, due to its ability to compete against other bacteria during sausage fermentation (Champomier‐Vergès, Chaillou, Cornet, & Zagorec, 2001; Hammes & Hertel, 1998; Leroy, Verluyten, & Vuyst, 2006; Zagorec & Champomier‐Vergès, 2017). Furthermore, Hufner and Hertel (2008) demonstrated that *L. sakei* retained its high performance in sausage fermentation even after sublethal stress treatments were applied prior to inoculation into the meat matrix. Moreover, Leroy and De Vuyst (1999, 2005) have demonstrated that the bacteriocin‐producing strain *L. sakei* CTC494 may represent both a promising protective and functional sausage starter culture. Consequently, in recent years, Tunisian dry‐fermented sausages manufactured using indigenous starter cultures have become an important focus of study (El Adab, Essid, & Hassouna, 2014; Essid & Hassouna, 2013; Mejri et al., 2017). Tolerance to bile salt is considered one of the essential properties required for lactic acid bacteria to survive in the small intestine environment (Succi et al., 2005). In some cases, the strains were known to have antipathogenic activity which is proposed as probiotic. Microbiological parameters were collected including total bacterial count, LAB count, numbers of enterobacteria, yeasts, molds, and the presence of the pathogens *Staphylococcus aureus* and *Salmonella* spp. Physicochemical parameters, including pH, water activity (aw), moisture content, and fatty acid profiles, were followed until 45 days of ripening, and the sensory profile of the finished product was compared with sausage produced using commercially available starter cultures. We demonstrate that the anchovy‐isolated strain *L. sakei* BMG 95 shows promise as a future starter strain based on its technological and antimicrobial characteristics.

Thus, the aim of the present study was to determine the quality characteristics of locally produced turkey dry‐fermented sausages using tow novel *L. sakei* strains as starter cultures.

## 2 MATERIALS AND METHODS

### 2.1 Bacterial strains and inocula preparation

Starter cultures consisted of the following LAB strains: *L. sakei* 23K, isolated from a French sausage (Chaillou et al., 2005); *L. sakei* BMG 95, isolated from anchovies; *L. sakei* BMG 37, isolated from sheep meat (Najjari, Ouzari, Boudabous, & Zagorec, 2008); and a *Staphylococcus xylosus* strain isolated from artisanal Tunisian fermented sausages (GenBank accession number: SUB7037332 XYLO MT111928). The commercial starter (RAPS, 730 Biostart plus) was used as a control starter strain for this study. *Lactobacillus sakei* strains were grown in MRS broth at 30°C for 24 hr, and *S. xylosus* was grown in BHI broth at 37°C for 24 hr. For inocula preparation, one liter of each culture was centrifuged at 14,515 rcf for 10 min at 4°C, and then, the pellet was washed and resuspended in sterile saline (0.9% NaCl, w/v). Sausage batters were inoculated using the following combinations of LAB together with *S. xylosus*: inoculant 1: *L. sakei* 23K + *S. xylosus*; inoculant 2: *L. sakei* BMG 37 + *S. xylosus*; inoculant 3: *L. sakei* BMG 95 + *S. xylosus* and inoculant 4: the commercial starter. Noninoculated sausages were also included as a control.

### 2.2 Technological properties of *L. sakei* starter cultures: inhibitory potential

#### 2.2.1 Antimicrobial assessment

The inhibitory effect of *L. sakei* strains on selected indicator bacteria (*Listeria innocua, Listeria ivanovii*, *Enterococcus faecalis*, *Staphylococcus aureus*, *Salmonella* spp.,* Bacillus cereus,* and *Escherichia coli 8739*) was determined using the well‐diffusion method described by Saavedra, Minahk, Holgado, and Sesma (2004). Briefly, an overnight culture of the indicator strain (approximately 10^6^ cells/ml) was spread onto the surfaces of Brain Heart Infusion (BHI) agar plates. Then, wells of 5 mm diameter were cut into the surface of the plates and 100 μl of filtered (0.22 µm) *L*. *sakei* culture supernatant was added to each well. The prepared plates were incubated for 24 hr at 30°C, and the antimicrobial activity was recorded as the diameter of inhibition zones observed around the well. Inhibition was recorded as negative if no clear zone was observed around the agar well. The antagonistic strains *L. sakei* LB674, producing sakacin P, and *L. sakei* Lb706, producing sakacin A (Axelsson & Holck, 1995), were used as positive indicators, while MRS broth was used as a negative control.

#### 2.2.2 Bile salt tolerance

Tolerance to bile salt was tested according to the method of Pennacchia et al. (2004) with some modifications. Briefly, *L. sakei* strains were grown in MRS broth at 30°C overnight. Saturated bile solution was filtered through 0.45 µm filter and was added to the cultures to a final concentration of 0.3%. A nontreated culture was included as a control. The cultures were incubated at 37°C for 3 hr. Viable counts of *Lactobacillus* strains were determined by pour plate counts of all the samples using 10‐fold serial dilutions prepared in 0.1% peptone water. All the experiments were in triplicate.

### 2.3 Sausage manufacturing process

Sausages were produced using frozen and raw turkey thigh meat (43% and 30%, respectively) and frozen beef fat (27%). Spices and other additives were included to a level of 43 g/kg. Frozen meat and fat were tempered to −2°C before use. After grinding the meat and fat to particle sizes of 6 mm in diameter, the spices and other ingredients were added. Meat and fat were then further reduced to pieces of 2 mm maximum in diameter by continuous mixing and homogenization. Fourteen kilograms of batter was prepared and divided into 5 equal parts. Four of the parts were inoculated using one of the four prepared inoculants 1–3, containing 10^6^ CFU/g and 10^3^ CFU/g of *L. sakei* and *S. xylosus,* respectively, reflecting the levels used by Coffey et al. (1998) and 4, containing the commercial starter. The fifth part was used as the uninoculated control sample. The mixture was stuffed in permeable sausage casings (diameter 45 mm) and equilibrated at room temperature for 2 days at 22°C and 90% relative humidity (RH), then cold smoked for 3 hr at 26°C, incubated for 5 days at 18°C and 80% RH, and dried for 1 hr at 60°C. Finally, sausages were ripened until day 24. During ripening, the temperature and relative humidity were gradually reduced to 14°C and 70%–80% RH, respectively. Sausages from each part were sampled at 0, 9, 24, and 45 days and samples then subjected to microbiological, physicochemical, nutritional, and sensorial analyses.

### 2.4 Microbiological analysis

Twenty‐five grams of each sample was taken aseptically, diluted in 225 ml of 0.1% peptone water, and homogenized for 2 min. After serial dilution, appropriate dilution samples (1 or 0.1 ml) were poured or spread on agar plates. Microbiological analysis was performed according to the International Organization for Standardization (ISO), “Association française de normalisation” (AFNOR) and “Norme française” (NF) norms. Total viable count (TVC) was determined on plate count agar (PCA), incubated at 30°C for 72 hr according to NF ISO 6887 method. LAB count was performed on MRS agar, incubated at 30°C for 72 hr (NF ISO 15214, 1998); yeast and molds on oxytetracycline‐glucose yeast extract agar (OGA), incubated at 25°C for 72 hr (NF V 08‐059, 2002); *Enterobacteriaceae* were enumerated on violet red bile glucose agar (VRBL), incubated at 37°C for 48 hr (NF ISO 4832, July 1991); *Staphylococcus aureus* on Baird‐Parker agar (BP), incubated at 37°C for 48 hr (AFNOR‐08‐057 and ISO 6887); anaerobic sulfate‐reducing bacteria (ASR) on tryptone sulfite neomycin (TSN), incubated anaerobically at 37°C for 48 hr (AFNOR‐V08‐010/06, 1982 and ISO 6887); Salmonella on Salmonella Shigella (SS) Agar at 37°C for 48 hr (AFNOR‐V08‐052, 1993 and NF ISO 6579).

### 2.5 Physicochemical analysis

Physicochemical analysis during ripening included measurement of pH, aw, and moisture. Potentiometric measurements of pH were made using a pin electrode of a pH meter inserted directly into the sausage (NF V 04‐408). Three independent measurements were made on each sample. Water activity measurements were carried out using a water activity meter (Aqualab) at 25°C. Moisture content was determined by oven drying the sample at 100°C to constant mass (NF V 04‐401).

### 2.6 Quantification of lipid fraction and characterization of fatty acid

NF V 04‐402 method was used for the extraction of lipids from sausages. Forty milliliters of hexane was added to 5 g of sample, and the mixture was incubated 2 hr at −20°C and filtered to eliminate water. The weight of fat present in sample was determinate after evaporation of hexane by a Rotavapor at 42°C and 270 mba. Fatty acids were characterized by gas phase chromatography (CPG) (ISO 5509‐1978). To determine ester content, 40 ml of methanol and 0.5 ml potassium hydroxide methanolic solution were added to lipid fraction prepared previously. After decantation, the organic fraction obtained was boiled, cooled, and rinsed with 20 ml of heptane. The last step was repeated twice. Finally, the heptane was evaporated par Rotavapor at 42°C and 60 mba, and the esters obtained was injected in CPG column.

### 2.7 Sensory evaluation

A sensory panel containing twelve trained members was used for the evaluation of dry‐fermented sausage samples after 24 days of ripening. Sausages were evaluated for texture, flavor, taste, and odor using a 6‐point Hedonic scale, ranging from 0 for very low intensity to 10 for very high intensity.

### 2.8 Statistical analysis

Significant differences between means of three replicates and standard deviations were determined using one‐way analysis of variance (ANOVA) performed with Origin 8 software (OriginLab® Origin 8.5.1). Tukey's range test was used to determine any significant difference between mean values, and evaluations were based on a significance level of *p* < .05.

## 3 RESULTS AND DISCUSSION

### 3.1 Technological properties of *L. sakei* starter cultures

#### 3.1.1 Antibacterial activity

Antibacterial activity of *L*. *sakei* strains was positive against the gram‐positive targeted bacteria *L.* *innocua*, *L. ivanovii*, *E. faecalis*, and *S. aureus,* as well as *Salmonella*. *Lactobacillus* *sakei* is known to produce several classes of bacteriocins with a wide range of characteristics such as *sakacin* known to be active against only gram‐positive taxa, including *Listeria* spp. and *Enterococcus* spp. (Axelsson, Katla, Bjørnslett, Eijsink, & Holck, 1998; Leroy & De Vuyst, 1999). These activities could prevent the colonization of gastrointestinal tract by pathogenic bacteria which is considered as a primary mechanism of beneficial effects mediated by probiotics properties.

#### 3.1.2 Ability to tolerate bile salts

Evaluation of the ability of *L*. *sakei* strains to survive in the presence of bile was determined by the method of decimal dilution and counting of CFU strains in the presence of 0.3% of bile salts. The results showed that all strains have a survival rate greater than 80%, ranging from 83%, 86%, and 89% for *L. sakei* 23K, *L. sakei* BMG 95, and *L. sakei* BMG 37, respectively. The concentration of bile salts used in this experiment (0.3%) was high in comparison to concentrations used in other studies, and it was used as tool for selection of potential LAB (Ibrahim & Bezkorovainy, 1993). This type of tolerance has been observed in other *Lactobacillus species* isolated from sausages (Klingberg & Budde, 2006; Pennacchia et al., 2004; Shin, Kang, Jang, & Kim, 2002). These results highlight the ability of these strains to survive the gastrointestinal environment.

### 3.2 Microbiological analyses

Fermented sausages were subjected to microbiological analysis to monitor the dynamic changes in the populations responsible for the ripening. Results of microbiological analysis of sausage produced with or without starter cultures of *L. sakei* and *S. xylosus* are presented in Table 1.

**TABLE 1:** Results of the microbiological analysis

Microbiological analysis parameter	Result (log_10_ CFU/g) for fermentation at day given^a^																			
	Fermentations																			
Strains	*L. sakei* 23K + *S. xylosus*				*L. sakei* BMG 37 + *S. xylosus*				*L. sakei* BMG 95 + *S. xylosus*				Commercial starter				Control			
Day of ripening	0	9	24	45	0	9	24	45	0	9	24	45	0	9	24	45	0	9	24	45
Total viable count	5.00 ± 0.67	5.12 ± 0.33	3.70 ± 0.24	3.85 ± 0.07	4.76 ± 0.05	4.61 ± 0.13	4.50 ± 0.70	4.63 ± 0.19	4.32 ± 0.38	4.48 ± 0.01	4.64 ± 0.18	4.78 ± 0.23	4.20 ± 0.34	3.56 ± 0.10	2.95 ± 0.21	3.00 ± 0.06	4.30 ± 0.67	4.95 ± 0.45	3.04 ± 0.32	5.00 ± 0.41
LAB	4.62 ± 0.46	5.04 ± 0.12	3.36 ± 0.15	3.60 ± 0.23	4.65 ± 0.14	5.32 ± 0.02	4.30 ± 0.10	4.70 ± 0.17	4.18 ± 0.66	4.66 ± 0.09	4.60 ± 0.24	4.80 ± 0.06	4.40 ± 0.42	4.75 ± 0.24	2.80 ± 0.03	3.30 ± 0.06	4.89 ± 0.12	5.78 ± 0.39	3.15 ± 0.32	3.79 ± 0.14
Yeasts	3.17 ± 0.05	3.04 ± 0.13	3.00 ± 0.10	4.84 ± 0.06	2.00 ± 0.04	2.93 ± 0.10	3.70 ± 0.36	3.95 ± 0.47	2.70 ± 0.13	2.25 ± 0.01	3.60 ± 0.09	4.70 ± 0.19	2.00 ± 0.02	2.32 ± 0.03	2.48 ± 0.18	3.50 ± 0.82	2.70 ± 0.29	3.01 ± 0.12	3.30 ± 0.25	5.49 ± 0.64
Molds	<10 N.A	<10 N.A	<10 N.A	<10 N.A	<10 N.A	<10 N.A	<10 N.A	<10 N.A	<10 N.A	<10 N.A	<10 N.A	<10 N.A	<10 N.A	<10 N.A	<10 N.A	<10 N.A	<10 N.A	<10 N.A	<10 N.A	<10 N.A
Total coliforms	2.70 ± 0.14	A	A	A	3.60 0.26	A	A	A	A	A	A	A	A	A	A	A	A	A	A	A
ASR	<50 N.A	<50 N.A	<50 N.A	<50 N.A	<50 N.A	<50 N.A	<50 N.A	<50 N.A	<50 N.A	<50 N.A	<50 N.A	<50 N.A	<50 N.A	<50 N.A	<50 N.A	<50 N.A	<50 N.A	<50 N.A	<50 N.A	<50 N.A
*S. aureus*	<100 N.A	<100 N.A	<100 N.A	<100 N.A	<100 N.A	<100 N.A	<100 N.A	<100 N.A	<100 N.A	<100 N.A	<100 N.A	<100 N.A	<100 N.A	<100 N.A	<100 N.A	<100 N.A	<100 N.A	<100 N.A	<100 N.A	<100 N.A
*Salmonella* spp.	A	A	A	A	A	A	A	A	A	A	A	A	A	A	A	A	A	A	A	A

Note

Values are expressed in log_10_ colony forming unit (cfu)/g.

Each number is the mean of two sausage samples taken from the same batch. Each sample was analyzed in duplicate.

Abbreviations: A, Absent in 25 g of product; N.A, not applicable; N.P, not performed.

^a^ Microbial growth at days 0, 9, 24 and 45 of maturation of the fermented sausages.

#### 3.2.1 Total viable count

The total viable count decreased slowly till the 24th day of ripening and did not exceed 10^5^ CFU/g in all sausages tested (Table 1). The relative decrease in the total flora at the end of maturation has been reported by other authors (Drosinos et al., 2004; Samelis, Metaxopoulos, Vlassi, & Pappa, 1998), after an increase during the first days of fermentation. Cocolin et al. (2009), showed an increase in the total flora in sausages sourced from five different producers and analyzed during the maturation cycle. This variation in the evolution of total flora in different sausages may be due to environmental conditions (Hammes, Bantleon, & Min, 1990).

In Canada, the Quebec center for the inspection of food and animal health (CQIASA) uses the standard MFHPB‐18, which deems sausages as unacceptable if the number of mesophilic aerobic bacteria exceeds 10^8^ CFU/g (Barthe, Daigle, Daigle, Desroches, & Roy, 2009). Our dry sausage sample measurements are thus in conformity with the acceptance criteria specified by the Quebec standard. Determining the TVC during the technological treatments makes it possible to judge the impact of various operations. However, a high TVC does not automatically correspond to an unacceptable product from a qualitative or hygienic perspective. A high level of bacteria may be compatible with a healthy product (e.g., fermented products), while small numbers may correspond to a hazardous product, if those numbers are composed of pathogenic bacteria or, even in the absence of bacterial counts, concerning levels active toxins produced by cells that may not have survived a given process (Guiraud & Rosec, 2004).

#### 3.2.2 Lactic acid bacteria

The number of lactic acid bacteria in day 0 of fermentation was between 10^4^ and 10^5^ CFU/g, which corroborates the observations of Comi et al. (2004), Rantsiou et al. (2004), and Samelis et al. (1998). The number of lactic acid bacteria always increases during fermentation of dry sausage. After fermentation and during maturation, however, the number of these microorganisms may decrease, increase, or stay constant. In our case, the number of lactic bacteria increased during the first nine days of fermentation and then decreased during the maturation process to stabilize at levels of around 10^3^–10^4^ CFU/g in all sausage samples (Table 1). However, such levels are acceptable under the CQIASA acceptability criteria 01‐M‐190, which deems sausages to be unacceptable (risk of alteration of the product), if the number of LAB exceeds 10^8^ CFU/g (Barthe et al., 2009). Studies by Rantsiou et al. (2004) and Drosinos et al. (2004) indicate a similar development of LAB populations in different batches of fermented dry sausage. In other cases, LAB populations have been shown to increase exponentially from day 0 to day 28 of dry‐sausage maturation (Comi et al., 2004). Such differences in LAB population development in fermented sausages made by different producers may be reasonable when the recipe and fermentation conditions used are taken into account (Rantsiou et al., 2004). Indeed, the development of lactic bacteria during the fermentation and maturation of dry sausage is likely influenced by a combination of factors including the manufacturing process, the ecological conditions within sausages, and the strains used for fermentation (Hammes, 1990).

#### 3.2.3 Yeasts and molds

Yeast counts on sausage samples increased from 10^2^ to 10^3^ CFU/g from day 0 to day 24 of maturation (Table 1). By day 45, the majority of samples showed stabilization of yeast counts with values around 10^4^ CFU/g. The control sample presented the highest yeast count (3 × 10^5^ CFU/g). However, samples inoculated by the commercial starter presented the lowest yeast counts compared to the four other samples inoculated by LAB. This suggests that *L. sakei* strains presented in commercial starter may have an inhibiting effect on the growth of yeasts. Among the principal yeast, genera found in dry sausage are *Candida*, *Debaryomyces,* and *Willopsis* (Rantsiou et al., 2004). Among yeasts, only a few species, such as *Candida albicans* and *Cryptococcus neoformans*, are pathogenic and the great majority do not cause human disease or food poisoning. However, lots of yeasts can cause plant diseases (Dean et al., 2012). Therefore, the presence of yeasts in many foods, although sometimes frequent, is not usually seen as a source of concern (Guiraud & Rosec, 2004). In contrast to yeasts, the mold counts observed in all the fermentations considered in the study were lower than 10 CFU/g.

#### 3.2.4 Other bacteria

In all samples, enterobacteria were only present at the beginning of fermentation, which was not unexpected as they are known for their sensitivity to acidic environments (Adams & Nicolaides, 1997). Zdolec et al. (2008) found similar results when they produced dry‐fermented sausage with bacteriocinogenic culture of *L. sakei* and semipurified bacteriocin mesenterocin Y. However, in contrast, our results did not support those of Cocolin et al. (2009), who demonstrated the persistence of enterobacteria until day 60 of maturation of dry‐fermented sausages, albeit at a low level. For all samples, levels of *S. aureus* and anaerobic sulfite‐reducing bacteria (ASRB) were lower than 100 CFU/g and 50 CFU/g, respectively. Drosinos et al. (2004) and Samelis et al. (1998) reported similar findings. AFNOR (Association Française de Normali*sation*) allow up to 5 × 10^2^ CFU/g and 50 UCF/g for *S. aureus* and ASRB, respectively, in acceptable sausage. Therefore, our results indicate that our tested processes conform to an acceptable standard. In contrast, *Salmonella* was absent during fermentation and ripening of sausages. Our results reinforce previous findings that *Salmonella* tends to be absent during the manufacture of dry‐fermented sausage (Cocolin et al., 2009; Comi et al., 2004; Drosinos et al., 2004; Rantsiou et al., 2004).

### 3.3 Water activity, pH and weight loss

During ripening of sausages, the water activity (*a_w_*) decreased (*p* < .05) from initial values ranging between 0.983 and 0.987 in the raw product to values of 0.849 and 0.824 at the 45th day of ripening (Table 2). The use of particular starter cultures was not associated (*p* > .05) with any differences observed in a_w_, supporting the earlier findings of Zapelena et al. (1999) and El Adab et al. (2014). Addition of salt and the drying process causes a decrease in a_w_ (Hugas & Monfort, 1997). The lowest a_w_ value detected in our study was detected in the sample inoculated with *L. sakei* BMG 95 and *S. xylosus*. *Lactobacillus sakei* BMG 95 was isolated from anchovy, so possibly, the osmotic stress triggered by high salt concentrations might not cause injury to this strain during the ripening process due to the presence of glycine–betaine components that could be used by the starter culture cells to remain robust in the low aw environment of salted anchovy based products (Belfiore, Fadda, Raya, & Vignolo, 2013). The decrease of water activity in traditional sausage is important for enhancing shelf life and safety (Chevallier et al., 2006). At day 9 storage, the initial pH had declined in both treated and control sausage samples (Table 2). However, by day 24, the pH of all samples had increased and was stable through to day 45 of maturation. The pH of inoculated sausages was significantly (*p < *.05) lower than for control sausages, with the lowest values reported in sausages inoculated with the commercial starter (Table 2). It seems evident that the higher pH observed in control sausages is related to the lower LAB counts observed in the same sausages, which were not inoculated with starter (Table 1). Conversely, the decline in pH in inoculated sausages correlated with increased proliferation of LAB in sausages. This observation supports the general observation that fermentation of carbohydrates by LAB is associated with generation of organic acids and decreasing pH (Samelis et al., 1998).

**TABLE 2:** Physical and chemical analysis of the fermented sausages used in this study during the fermentation and ripening process

Strains	Fermentations																				Sign.^a^
	*L. sakei* 23K + *S. xylosus*				*L. sakei* BMG.37 + *S. xylosus*				*L. sakei* BMG.95 + *S. xylosus*				Commercial starter				Control				
Day of ripening	0	9	24	45	0	9	24	45	0	9	24	45	0	9	24	45	0	9	24	45	
Physical analysis																					
pH	6.24 ± 0.08	5.41 ± 0.02	5.59 ± 0.13	5.53 ± 0.06	6.21 ± 0.04	5.57 ± 0.01	5.84 ± 0.03	5.81 ± 0.01	6.10 ± 0.05	5.46 ± 0.02	5.58 ± 0.01	5.50 ± 0.03	6.16 ± 0.07	5.42 ± 0.01	5.48 ± 0.02	5.57 ± 0.05	6.17 ± 0.04	5.51 ± 0.03	5.58 ± 0.03	5.55 ± 0.01	*
*Aw*	0.98 ± 0.08	0.92 0.00	0.89 ± 0.01	0.84 ± 0.01	0.98 ± 0.02	0.90 0.00	0.88 0.00	0.85 ± 0.05	0.98 ± 0.01	0.91 ± 0.01	0.88 ± 0.02	0.82 ± 0.06	0.98 ± 0.02	0.88 ± 0.02	0.88 ± 0.01	0.85 ± 0.05	0.97 ± 0.01	0.91 0.00	0.88 ± 0.03	0.84 ± 0.02	NS
RH (%)	62.49 ± 2.02	39.24 ± 0.54	35.3 ± 0.39	32.00 ± 0.11	63.16 ± 0.99	38.49 ± 0.34	35.86 ± 0.55	34.50 ± 0.31	62.64 ± 0.23	32.53 ± 0.51	33.07 ± 0.47	32.53 ± 0.58	66.16 ± 1.65	42.19 ± 0.85	34.39 ± 0.35	35.41 ± 0.42	63.26 ± 0.53	44.38 ± 1.04	37.87 ± 0.24	33.35 ± 0.41	NS
Chemical analysis																					
Fat (%)	35.10 ± 0.71	14.15 ± 0.39	35.10 ± 0.04	22.50 ± 0.10	40.66 ± 1.09	19.14 ± 0.15	29.62 ± 1.00	17.94 ± 0.02	18.36 ± 0.84	18.44 ± 0.01	26.47 ± 0.94	23.38 ± 0.40	21.77 ± 0.12	17.27 ± 0.08	14.33 ± 0.07	17.55 ± 0.18	21.96 ± 0.36	28.44 ± 1.25	26.44 ± 0.06	19.90 ± 0.03	NS

Abbreviation: NS, not significant.

^a^ Significance.

* Significant at *p < *.05.

For all sausages samples, moisture content decreased throughout the ripening process from initial values of around 65 to around 35% (Table 2) supporting similar findings made by other workers (El Adab et al., 2014; Essid & Hassouna, 2013; Zapelena et al., 1999). Hoz, D'Arrigo, Cambero, and Ordonez (2003) explained that moisture loss is a consequence of progressive and partial dehydration of the meat. However, like the final fat content, which was around 14%–46%, water activity and moisture appear to be factors not related to the choice of starter strain.

### 3.4 Fatty acid profile

There were no significant differences between the proportions of fatty acids obtained throughout the days of ripening (Table 3). Consequently, the process of fermentation does not appear to affect the proportion and composition of fatty acids in sausage. Saturated (SFA), monounsaturated (MUFA), and polyunsaturated (PUFA) fatty acids were present in, respectively, proportions of 40%, 50%, and 10% (Table 4), a characteristically common distribution of fatty acids in meat (Wood, Enser, Richardson, & Whittington, 2008). Oleic acid (C18:1) was present with an average proportion of 40% of total fatty acids, reflecting prior observations that oleic acid constitutes the majority fatty acid in all meats (Wood et al., 2008). Sausage inoculated with *L. sakei* BMG 95 and *S. xylosus* and sausage inoculated with the commercial starter showed a significant (*p* < .05) increase in PUFA, 17.60% and 16.16%, respectively, compared to their corresponding control (12.64%) at the 24th day of maturation (Table 4). This suggests that strains of *Lactobacillus* and *Staphylococcus* used as a ferment in these samples could potentially possess lipolytic activity, which could explain the increased PUFA proportions in meat, supporting similar observations made by Zalacain, Zapelena, Astiasarán, and Bello (1995) and Arief, Afiyah, Wulandari, and Budiman (2016).

**TABLE 3:** Fatty acid composition of dry sausage along the fermentation cycle (g/100 g of fatty acids)

Strains	Fermentations																			
	*L. sakei* 23K + *S. xylosus*				*L. sakei* BMG.37 + *S. xylosus*				*L. sakei* BMG.95 + *S. xylosus*				Commercial starter				Control			
Day of ripening	0	9	24	45	0	9	24	45	0	9	24	45	0	9	24	45	0	9	24	45
SFA																				
C12:0	0.20 0.00	1.74 ± 0.01	0.12 0.00	0.10 ± 0.01	3.50 ± 0.11	0.34 ± 0.05	0.08 ± 0.01	0.10 0.00	0.10 0.00	0.12 0.00	0.20 ± 0.03	0.10 ± 0.01	0.13 ± 0.01	0.43 ± 0.12	0.10 0.00	0.14 0.00	0.07 0.00	0.09 ± 0.01	0.10 0.00	0.10 ± 0.02
C14:0	1.90 ± 0.01	2.97 ± 0.24	1.97 ± 0.14	2.47 ± 0.04	2.24 ± 0.03	2.50 0.00	1.88 ± 0.02	2.21 0.00	2.10 ± 0.05	2.40 ± 0.01	1.80 ± 0.04	2.34 0.00	2.60 0.00	2.22 ± 0.02	2.00 ± 0.01	2.38 ± 0.06	2.11 ± 0.04	2.00 ± 0.11	2.16 ± 0.09	2.13 ± 0.12
C16:0	18.4 ± 0.34	19.49 ± 0.04	18.74 ± 0.10	22.92 ± 0.03	17.12 ± 0.11	21.00 ± 0.21	19.23 0.00	20.69 ± 1.03	20.00 ± 0.02	20.00 0.00	16.91 ± 0.62	20.72 ± 1.12	23.00 ± 2.35	19.94 ± 0.14	18.84 ± 0.05	21.00 0.00	20.60 ± 1.01	20.60 ± 0.63	19.00 ± 1.14	21.38 ± 2.03
C17:0	0.65 ± 0.02	0.69 ± 0.10	0.81 ± 0.04	1.10 0.00	0.60 0.00	0.89 ± 0.08	0.85 ± 0.05	0.84 0.00	0.85 ± 0.01	0.80 ± 0.03	0.57 0.00	0.92 ± 0.04	1.00 ± 0.21	0.73 0.00	0.64 ± 0.07	0.88 0.00	1.00 ± 0.23	0.78 ± 0.42	0.80 0.00	0.91 ± 0.08
C18:0	10.00 ± 0.01	10.71 ± 0.04	11.90 ± 0.24	18.52 ± 1.64	8.37 ± 4.52	13.82 ± 1.32	13.64 ± 0.09	12.87 ± 0.41	13.60 ± 0.23	12.00 ± 0.08	9.00 ± 3.05	13.67 ± 0.14	16.48 ± 1.95	11.66 ± 0.56	9.29 ± 1.15	13.29 ± 0.06	16.68 ± 0.17	12.84 ± 0.02	11.66 ± 2.31	14.96 ± 0.14
C20:0	0.34 0.00	0.35 ± 0.01	0.39 0.00	4.30 0.00	0.44 ± 0.11	0.31 ± 0.05	0.32 ± 0.02	0.30 0.00	0.30 ± 0.01	0.35 ± 0.04	0.36 ± 0.00	1.14 ± 0.06	0.29 0.00	0.28 ± 0.08	0.32 ± 0.01	0.30 ± 0.03	0.36 ± 0.07	0.29 0.00	0.45 ± 0.01	0.27 0.00
MUFA																				
C16:1	3.82 ± 0.20	3.62 ± 0.11	3.46 ± 0.04	2.75 ± 0.01	4.18 ± 0.16	3.32 ± 0.03	3.00 ± 0.01	3.40 0.00	3.32 ± 0.06	3.49 ± 0.01	4.00 ± 0.61	3.00 ± 0.02	3.18 ± 0.10	3.77 ± 0.03	4.40 ± 0.31	3.53 0.00	3.00 ± 0.35	3.40 ± 0.01	3.62 ± 0.22	3.22 ± 0.10
C17:1	0.63 0.00	0.65 ± 0.02	0.77 ± 0.03	0.55 0.00	0.76 ± 0.05	0.61 0.00	0.60 0.00	0.64 ± 0.01	0.64 0.00	0.73 ± 0.04	0.70 0.00	0.63 ± 0.01	0.60 0.00	0.59 ± 0.03	0.70 ± 0.02	0.68 ± 0.01	0.63 ± 0.01	0.53 0.00	0.71 0.00	0.57 ± 0.08
C18:1 (w‐9c)	44.58 ± 0.51	38.75 ± 1.04	45.55 ± 0.09	34.58 ± 1.83	40.53 ± 2.00	38.34 ± 0.05	40.66 ± 0.01	42.16 ± 0.05	38.96 ± 0.71	42.62 ± 0.40	43.95 ± 0.06	38.84 ± 0.14	35.19 ± 0.08	39.52 ± 1.06	43.28 ± 1.09	39.00 ± 0.60	39.27 ± 0.09	38.13 ± 0.04	42.97 ± 0.92	37.45 ± 0.63
C18:1 (11 cis)	2.00 0.00	2.42 ± 0.02	1.97 ± 0.31	2.00 ± 0.01	2.03 0.00	2.14 ± 0.05	2.28 0.00	2.00 0.00	2.00 0.00	2.64 ± 0.42	2.25 0.00	2.13 0.00	1.90 ± 0.11	2.64 ± 0.03	2.32 0.00	2.00 ± 0.01	2.00 0.00	2.36 ± 0.04	2.00 ± 0.06	2.94 ± 0.03
C20:1	0.13 ± 0.02	0.09 0.00	0.13 ± 0.01	0.17 0.00	0.33 0.00	0.15 ± 0.03	0.15 0.00	0.10 0.00	0.13 0.00	0.10 0.00	0.09 0.00	0.05 ± 0.01	0.14 0.00	0.10 0.00	0.08 0.00	0.13 ± 0.01	0.17 0.00	0.12 0.00	0.10 0.00	0.13 ± 0.03
PUFA																				
C18:2 (w‐6c)	0.11 ± 0.03	0.00 0.00	0.22 ± 0.02	0.00 0.00	1.00 0.00	0.21 ± 0.01	0.00 0.00	0.24 0.00	0.21 0.00	0.30 ± 0.04	0.20 ± 0.03	0.00 0.00	0.19 ± 0.01	0.00 0.00	0.32 ± 0.03	0.20 0.00	0.16 0.00	0.00 0.00	0.14 0.00	0.27 ± 0.04
C18:3 (w‐3)	1.30 ± 0.01	1.15 0.00	1.00 0.00	0.76 ± 0.01	1.34 ± 0.03	0.97 0.00	0.83 ± 0.02	1.00 ± 0.01	0.93 ± 0.03	0.82 ± 0.03	1.21 ± 0.10	1.46 ± 0.12	0.90 0.00	0.98 0.00	1.00 0.00	0.92 ± 0.01	0.80 ± 0.03	1.00 0.00	0.98 ± 0.01	0.76 ± 0.04
TFA																				
C18:1n9t	0.00 0.00	3.26 ± 0.02	0.00 0.00	3.90 ± 0.14	2.97 ± 0.03	2.78 ± 0.04	3.80 0.00	0.00 0.00	2.66 ± 0.15	2.50 ± 0.01	2.35 0.00	3.74 ± 0.31	3.00 ± 0.01	2.69 ± 0.05	1.78 ± 1.01	2.45 ± 0.01	3.15 ± 0.06	3.34 ± 0.23	3.64 0.00	4.15 ± 0.14
C18:2 (w‐6t)	15.88 ± 0.94	14.21 ± 1.06	12.98 ± 2.01	10.00 ± 2.42	4.63 ± 2.50	12.59 ± 0.13	12.60 ± 0.05	13.36 ± 0.41	12.15 ± 0.71	11.00 ± 0.02	16.20 ± 0.09	11.17 ± 0.04	11.30 0.00	14.45 ± 0.03	14.84 ± 0.62	12.97 ± 2.31	9.91 ± 0.37	14.42 ± 0.06	11.52 ± 1.13	10.75 ± 0.34

Note

Values are the mean of three replicates; standard deviations are in the range [±0.01 to ±4.52].

**TABLE 4:** Lipid fractions and nutritional values of interest in dry sausage along the fermentation process

Strains	*L. sakei* 23K +* S. xylosus*				*L. sakei* BMG.37 + *S. xylosus*				*L. sakei* BMG.95 + *S. xylosus*				Commercial starter				Control			
Day of ripening	0	9	24	45	0	9	24	45	0	9	24	45	0	9	24	45	0	9	24	45
Σ SFA	31.49	35.95	33.93	49.41	32.27	38.86	36	37.04	36.95	35.67	28.84	38.89	43.5	35.26	31.19	38	40.82	36.6	34.17	39.75
Σ MUFA	51.16	48.7	51.88	43.95	50.8	47.34	50.49	48.3	47.71	52	53.34	48.39	44	49.31	52.56	47.79	48.22	47.88	53	48.46
Σ PUFA	17.29	15.36	14.2	10.76	16.97	13.77	13.43	14.6	13.29	12.12	17.6	12.63	12.39	15.43	16.16	14	10.87	15.42	12.64	11.78
PUFA/SFA	0.55	0.4	0.4	0.2	0.5	0.35	0.4	0.4	0.36	0.34	0.6	0.3	0.3	0.4	0.5	0.4	0.3	0.4	0.4	0.3
n‐6/n‐3	12.3	12.36	13.2	13.16	11.7	13	15	13.6	13.3	14	13.5	7.6	13	14.7	15	14.3	12.6	14.42	12	14.5

The two main parameters commonly used to evaluate the nutritional value and healthfulness of lipid fractions in foods are PUFA/SFA and n‐6/n‐3 ratios (Burlingame, Nishida, Uauy, & Weisell, 2009; Griffin, 2008). Nowadays, the ratio of PUFA/SFA is recommended to be between 0.4 and 1 (Enser, Richardson, Wood, Gill, & Sheard, 2000; Wood et al., 2004). In our results, this ratio varied from 1:10 (0.1) to 1:1.7 (0.6) with a highest ratio (0.6) observed at day 24 of maturation in the samples inoculated with *L. sakei* BMG 95 and *S. xylosus*. This sample therefore contained a lipidic fraction with a nutritional quality that conformed with the recommendations established by Enser et al. (2000) and Wood et al. (2004). Probably, *L. sakei* BMG 95 can influence the increasing of PUFA proportion. According to the recommendations established by Enser et al. (2000) and Wood et al. (2004), the ratio n‐6/n‐3 should not exceed the value 0.4 (1:2.5). There is no significant (*p* > .05) change in this ratio throughout the fermentation (Table 4). Ferments used therefore had no effect on the proportions of α‐linolenic acid (C18: 3) and linoleic acid (C18: 2). The mean n‐6/n‐3 ratio is 13, close to the estimated n‐6/n‐3 ratio in the Western Spain diet and in the control dry‐sausage sample of the study of Ansorena and Astiasarán (2003). In addition, the *L. sakei* BMG 95 and *S. xylosus* inoculated sample is the only sample with an n‐6/n‐3 ratio = 7.6 close to the ratio required in the recommendations. This sample shows the highest proportion (1.46%) of α‐linolenic acid (C18: 3) at day 45 of fermentation (Table 3). This suggests that *L. sakei* BMG 95 strain may have a lipolytic activity against long carbon chain triglycerides, which has resulted in the release of PUFA such as α‐linolenic acid and an increase in its level in meat. Moreover, a high level of α‐linolenic acid and a low n‐6/n‐3 ratio can exert a suppressive effect on the development of certain pathologies such as cardiovascular diseases, cancers, and inflammatory and autoimmune diseases (Goodstine et al., 2003; Gutiérrez, Svahn, & Johansson, 2019; Marventano et al., 2015). On the other hand, an excessive level of linoleic acid (C18: 2) and a high n‐6/n‐3 ratio may promote the development of these chronic diseases (Simopoulos, 2008). Moreover, it has been suggested that risk factors for arteriosclerosis and myocardial ischemia may be less related to hypercholesterolemia than they are to a high n‐6/n‐3 ratio (Okuyama, Fujii, & Ikemoto, 2000). Thus, the n‐6/n‐3 ratio in the diet should ideally be balanced for normal growth and to reduce risks associated with cardiovascular and other chronic diseases.

### 3.5 Sensory analysis

**FIGURE 1:**
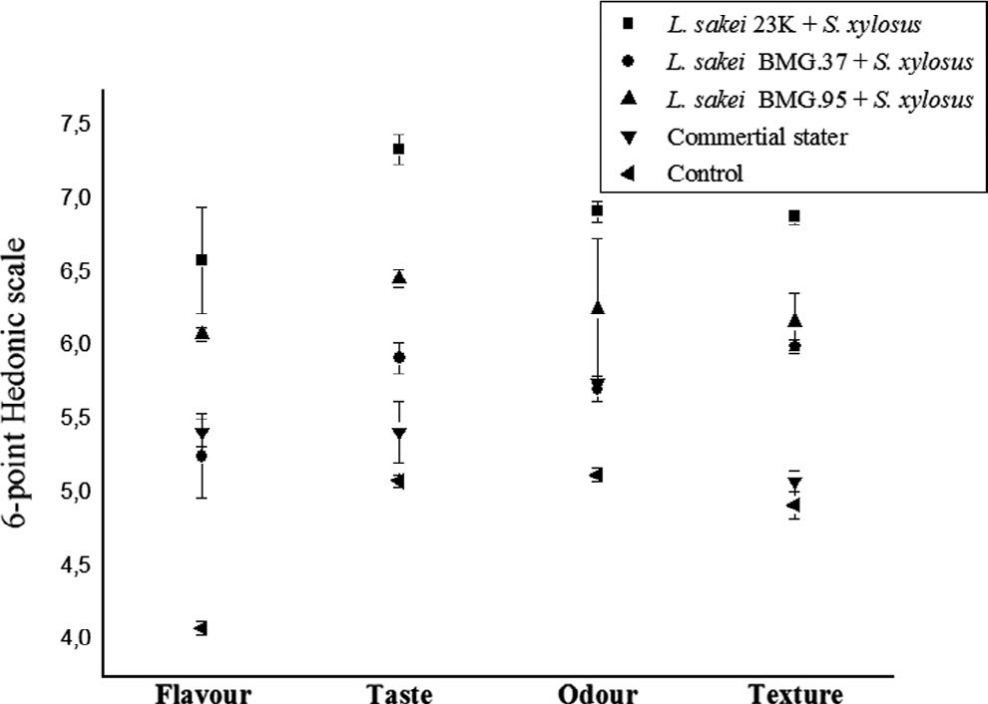
Sensory evaluation of starter‐inoculated and control dry‐fermented sausages considered in the study. Mean values corresponding to the same sensory parameter evaluated group not having a common letter differ significantly (*p* < .05)

Sensory analysis of dry‐fermented sausage was performed for all sausage samples (Figure 1). Results of the Tukey test revealed several differences among samples when comparing flavor, taste, and fuller odor mean values, and the ANOVA performed on the sensory results showed that these sensory parameters were significantly (*p < *.05) affected by the addition of LAB and commercial starter cultures, with the lowest values corresponding to the control sausage samples. These findings support similar conclusions made by Lorenzo, Gómez, Purriños, and Fonseca (2015). Interestingly, samples inoculated with *L.* sakei 23K and *S. xylosus* and samples inoculated with *L. sakei* BMG 95 and *S. xylosus* appear to have the best potential for producing a dry‐fermented sausage that is more aromatized with taste and flavor, more intense compared to the ones inoculated with *Lb. sakei* BMG37 and *S. xylosus*, or with the commercial starter or the noninoculated control sausages. It is known that coagulase‐negative staphylococci are used in the fermentation of dry sausage for their ability to produce a product characterized by a better flavor (Montel et al., 1998; Stahnke, Holck, Jensen, Nilsen, & Zanardi, 2002). Furthermore, *L. sakei* is characterized by a high enzymatic potential, responsible for the development of flavor, taste, and odor of meat products (Papamanoli, Tzanetakis, Litopoulou‐Tzanetaki, & Kotzekidou, 2003). Champomier‐Vergès et al. (2001) demonstrated that inoculation of meat by cellular extract of *L. sakei* was associated with an accumulation of free amino acids due to proteolysis. Thus, free amino acids such as glutamic acid and alanine appear to participate in the development of flavor of meat products (Champomier‐Vergès et al., 2001). These attributes also appear to be related to a range of other compounds, such as fatty acids, peptides, amino acids, aldehydes, and esters released by microbial and endogenous proteases and lipases throughout ripening of sausages (Essid & Hassouna, 2013; Mejri, Vásquez‐Villanueva, Hassouna, Marina, & García, 2017).

The texture of dry‐fermented sausages, inoculated with LAB, showed no significant differences with the control (Figure 1). This outcome is similar to that observed by El Adab et al. (2014). However, hardness and elasticity were affected by ripening times. Lorenzo et al. (2015) have suggested that hardness could arise from microbiological and physicochemical processes such as enhanced acidification and proteolysis of *L. sakei*, *S. xylosus,* and *Staphylococcus carnosus* activities. It could also be due to much higher weight loss during fermentation (Liaros, Katsanidis, & Bloukas, 2009).

## 4 CONCLUSION

Microbiological analyses of Tunisian dry‐fermented sausage show that no pathogenic or other quality‐reducing strains were detected in the dominant microbial community during the maturation process. The development of populations of different competitive starter cultures during the process of fermentation can be seen to be associated with the repression of such organisms due to competitive growth of LAB resulting in acidification of the product. Sausage samples inoculated with *L. sakei* BMG 95 showed the most desirable physicochemical and sensory attributes compared to samples inoculated with a commercial starter culture. Therefore, this strain, isolated from anchovies, has the potential for use as a starter culture in the commercial production of dry‐fermented sausage.

## CONFLICT OF INTEREST

The authors declare that they do not have any conflict of interest.

## ETHICAL APPROVAL

Production of this product was done in sterile condition, and before consumption by panellist, microbial analysis was evaluated. Sensorial evaluation was performed in test panellist room. Furthermore, this work do not involves the use of animals.

## INFORMED CONSENT

Written informed consent was obtained from all study participants.
